# Investigating the Properties of Electrodeposited of Ni-P-ZrC Nanocomposite Coatings

**DOI:** 10.1021/acsomega.1c03117

**Published:** 2021-12-01

**Authors:** Osama Fayyaz, A. Bahgat Radwan, Mostafa H. Sliem, Aboubakr Moustafa Abdullah, Anwarul Hasan, R. A. Shakoor

**Affiliations:** † Center for Advanced Materials (CAM), Qatar University, 2713 Doha, Qatar; ‡ Department of Mechanical and Industrial Engineering, College of Engineering, Qatar University, 2713 Doha, Qatar

## Abstract

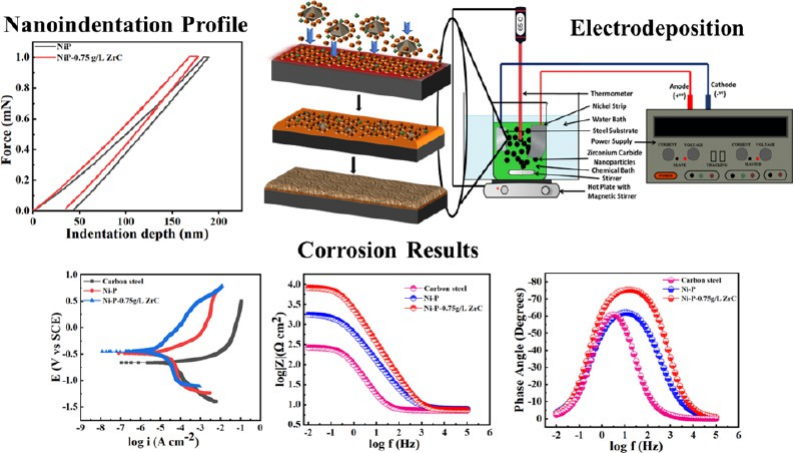

Superior corrosion resistance along with higher mechanical performance is becoming a primary requirement to decrease operational costs in the industries. Nickel-based phosphorus coatings have been reported to show better corrosion resistance properties but suffer from a lack of mechanical strength. Zirconium carbide nanoparticles (ZCNPs) are known for promising hardness and unreactive behavior among variously reported reinforcements. The present study focuses on the synthesis and characterization of novel Ni-P-ZrC nanocomposite coatings developed through the electrodeposition technique. Successful coelectrodeposition of ZCNPs without any observable defects was carried out utilizing a modified Watts bath and optimized conditions. For a clear comparison, structural, surface, mechanical, and electrochemical behaviors of Ni-P and Ni-P-ZrC nanocomposite coatings containing 0.75 g/L ZCNPs were thoroughly investigated. The addition of ZCNPs has a considerable impact on the properties of Ni-P coatings. Enhancement in the mechanical properties (microhardness, nanoindentation, wear, and erosion) is observed due to reinforcement of ZCNPs in the Ni-P matrix, which can be attributed to mainly the dispersion hardening effect. Furthermore, corrosion protection efficiency (PE%) of the Ni-P matrix was enhanced by the incorporation of ZCNPs from 71 to 85.4%. The Ni-P-ZrC nanocomposite coatings provide an exciting option for their utilization in the automotive, electronics, aerospace, oil, and gas industry.

## 1 Introduction

Corrosion can be regarded as a slow poison for metals and numerous alloys, thus affecting various industries, namely, water treatment plants,^1^ onshore pipelines,^2,3^ offshore pipelines,^3,4^ steelmaking processes,^5^ refineries,^6,7^ oil and gas industries,^4,7^ concrete structures,^8,9^ arthroplasty,^10,11^ storage tanks,^12^ geothermal equipment,^4^ biomedical devices,^13^ automobiles,^14^ microelectronics,^7,14^ textile industry,^15^ and aerospace and aeronautical applications.^16−18^ Adverse effects of corrosion and erosion due to harsh operating conditions have set difficult challenges and thus must be timely addressed, leading to obtaining various smart solutions.^19−21^ It is worth mentioning that the corrosion cost reaches 3–4% of the developed countries’ GDP.^22^ Moreover, the estimated cost of the metal deterioration in oil and gas is 170 billion USD per year.^22^ The corrosion risk can be extended to health and the environment due to failure in oil and gas equipment as a result of the wall thinning of the pipelines. Accordingly, many strategies are developed to mitigate corrosion in a harsh atmosphere, such as organic coatings, corrosion inhibitors, and metallic-based coatings.

Surface engineering of metal–metal alloys using metallic coatings grabbed researchers’ attention because of their promising corrosion solution and improved mechanical strength.^20,23−25^ Among inorganic coatings, nickel-based coatings are famous for their improved corrosion resistance and superior mechanical properties.^26−29^ Furthermore, the concept of composites is applied in nickel-based coatings to enhance their wear and erosion capabilities.^21,29,30^ Nickel-based coatings are synthesized through various methods, namely, direct current electrodeposition, pulse current electrodeposition, and electroless deposition.^26,31−33^ Direct current electrodeposition of a nickel-based composite coating is utilized by researchers due to its simplicity, stability of chemical bath, ease of tailoring the chemical bath, ease of scaling up and modifying the microstructure, and being economically feasible.^34^ Various aspects of electrodeposition are extensively studied, such as current density, pH, effects of additives, the effect of change in temperature, and deposition time to tailor the properties of coatings for different applications.^26,35−39^

The production and characterization of nanoscale species have provided the research community with opportunities to explore attractive improvements in the properties of various matrix materials.^40^ Reinforcing the Ni-P matrix with hard ceramics to improve its mechanical performance and enhancing its corrosion resistance ability are a comparatively novel idea that becomes tempting when the reinforcements are at the nanoscale. This reinforcing of hard ceramic nanoparticles improves the mechanical strength of the Ni-P coating.^41,42^ Enhancement in the characteristics of the Ni-P matrix has been obtained by considering numerous reinforcements as reported in the literature such as SiC,^42^ TiO_2_,^43^ C_3_N_4_,^44^ and WC,^45^ ZrO_2_,^46^ TiC,^47^ etc.

The main objective of the current research is to reinforce the Ni-P matrix with ZCNPs through an electrodeposition technique to develop novel Ni-P-ZrC metallic coatings and explore their impact on its structural, mechanical, wear, erosion, and corrosion characteristics. As per our literature survey, the synthesis of Ni-P-ZrC nanocomposite coatings through an electrodeposition technique and their characterization have not been previously reported. It is observed that the incorporation of ZCNPs into the Ni-P matrix has a significant effect and thus has resulted in remarkable development in properties such as corrosion, mechanical, erosion, and wear. The superior characteristics of Ni-P-ZrC alloys can be attributed to the combination of various factors such as (i) the dispersion hardening effect, (ii) blocking of pores using the inert ZCNPs, and (iii) reduction of active sites existing in the Ni-P matrix.

## 2 Materials and Methodology

### 2.1 Materials

The tailored chemical bath was modified from a Watts bath containing nickel chloride hexahydrate, nickel sulfate hexahydrate, sodium chloride, orthophosphoric acid, boric acid, and sodium hypophosphite monohydrate. Zirconium carbide (ZrC) nanopowder of <80 nm average size with a purity of 99.9% was purchased from Sigma Aldrich and used in the chemical bath. Nickel sheets and mild steel sheets were locally purchased to be used as the anode and cathode.

### 2.2 Sample Preparation

**Figure 1:**
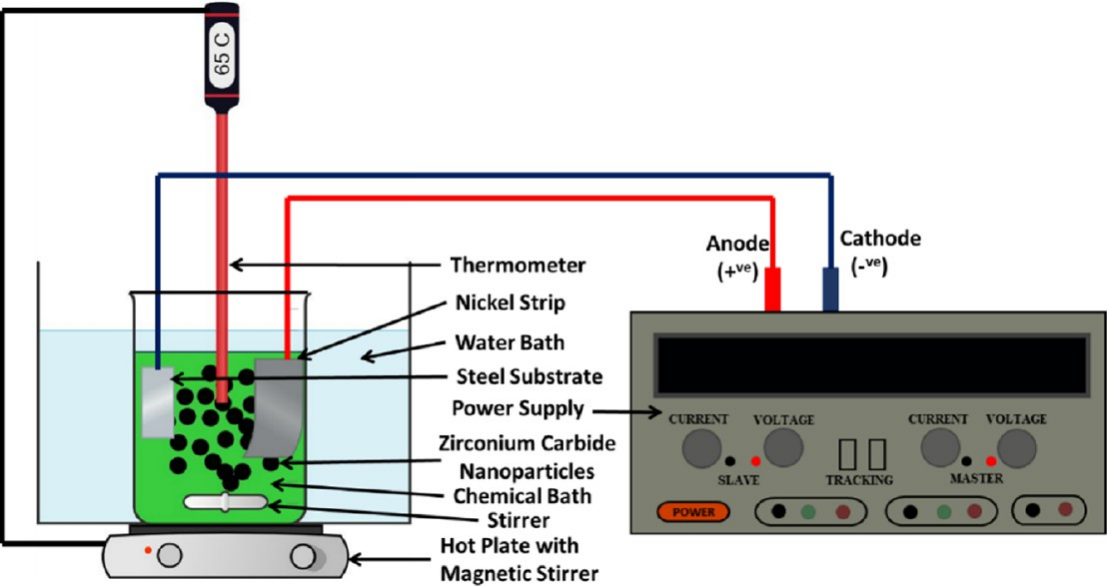
Schematic diagram of the employed electrodeposition process.

Synthesis of Ni-P-ZrC nanocomposite coatings was accomplished on polished mild steel substrates. In the first step, steel was made to a size of 32 mm coupons by metal sheet operation. Coupons were ground to produce polished samples on abrasive papers (silicon carbide) with gradings of 120, 220, 320, 500, 800, 1000, and 1200. The samples were washed with soap and water. Sonication with acetone was carried out after polishing for 25 min. In order to prevent electrodeposition on both sides of the substrates, tape was used to cover the unpolished sides. The steel specimens were etched in a 15% HCl solution for 40 s and rinsed in warm distilled water before placing it in the coating bath. The schematic representation of the electrodeposition experimental system is represented in Figure 1.

During the electrodeposition process, a nickel sheet was the anode, the polished steel substrate was the cathode, and they were placed parallel to each other at a distance of ∼30 mm in the chemical bath. The electrodeposition of Ni-P and Ni-P-ZrC (0.75 g/L) nanocomposite coatings was carried out at 65 ± 2 °C. The deposition time was fixed to be 30 min from the initiation of the power supply. In order to thoroughly disperse and prevent the settling down of the ZCNPs, the chemical bath was stirred at 300 ± 5 rpm before an hour of initiating electrodeposition. The tailored chemical bath and optimized deposition conditions are listed in Table 1.

**Table 1:** Tailored Chemical Bath Composition and Optimized Deposition Conditions for Coelectrodeposition of Ni-P and Ni-P-ZrC Nanocomposite Coatings

chemical bath and deposition conditions	Ni-P-ZrC
nickel sulfate hexahydrate	250 g L^–1^
nickel chloride hexahydrate	15 g L^–1^
boric acid	30 g L^–1^
sodium chloride	15 g L^–1^
orthophosphoric acid	6 g L^–1^
sodium hypophosphite monohydrate	20 g L^–1^
ZrC nanoparticles (<80 nm)	0 and 0.75 g L^–1^
pH	2.0 ± 0.2
temperature	65 ± 2 °C
deposition time	30 min
current density	48 mA cm^–2^
bath agitation	300 rpm

### 2.3 Sample Characterization

Structural characterization of the sample was done utilizing an X-ray diffractometer (Rigaku. Miniflex2 Desktop, Tokyo, Japan) employing Cu Kα radiations with a step size of 0.02° in the range of 2θ from 10 to 90°. The morphological study of the developed coatings was explored using a field-emission scanning electron microscope (FE-SEM; Nova Nano-450, Netherlands). A topographical study was carried out through an atomic force microscopy (AFM-USA) device MFP-3D from Asylum Research (USA). The equipment containing a silicon probe (Al reflex coated Veeco model OLTESPA, Olympus; spring constant, 2 N m^–1^; resonant frequency, 70 kHz) was used in these tests. All AFM investigations were performed at room temperature using tapping mode in air. Mechanical properties of the samples were tested on a Vickers microhardness tester (FM-ARS9000, USA) and an MFP-3D nanoindenter coupled with an AFM. The measurement of the microhardness was conducted at 25 gf with a dwell time of 10 s. The nanoindentation was evaluated using a Berkovich tip equipped with a diamond indenter bearing a maximum indentation load of 1 mN. More details about testing can be found elsewhere at refs (48) and (49). During wear testing, the sliding velocity was kept constant at a rate of 0.11 m s^–1^, keeping the constant diameter of the wear scar of 4 mm. The nanocomposite coatings acted as the disc and stainless-steel balls as a sliding medium. This test was carried out at 25 °C under 4 N normal loads and with a total sliding distance of 55 m. Erosion testing was done for the as-synthesized nanocomposite coatings using an air-jet erosion tester. Alumina particles were employed as an erodent as it is commonly used for corrosion testing. The particle size of the as-received alumina (Al_2_O_3_) is in the range of 53–84 μm. The experimental setup for performing the erosion tests followed the ASTM G76.^50,51^ The erodent particles flowed with a 0.94 g min^–1^ feed rate and were then ejected from the nozzle with a velocity range from 19 to 101 m s^–1^. The nozzle diameter is 2 mm, and the particle speeds were calculated based on the double-disc approach as Ruff and Ives presented a brief elucidation for calculating the particle speed by directly adjusting the gas pressure. The working distance between the nozzle outlet and the test specimen is 10 mm. The coating sample was mounted on a sample holder facing the nozzle with a 90° incident angle for different exposure times to achieve the maximum effect of surface deformation and depth. The depth and volume loss measurements for the exposed specimens were done using a 3D optical surface metrology system Leica DCM8 profilometer. The corrosion resistance of all the as-synthesized Ni-P and Ni-P-ZrC nanocomposite coatings was investigated using a Gamry 3000 potentiostat/galvanostat/ZRA (Warminster, PA, USA). A saturated silver/silver chloride electrode (Ag/AgCl) was utilized as per reference; however, graphite was used as a counter electrode, and the synthesized coating was employed as a working electrode. At an open-circuit potential, EIS spectra were determined by the AC signal at an amplitude of 10 mV within the 10^5^–10^–2^ Hz frequency range. Furthermore, the Tafel experiments were conducted at 25 °C using a scan rate of 1 mV s^–1^. An area exposure of 0.785 cm^–2^ of the synthesized coating was maintained during all the corrosion measurements.

## 3 Results and Discussion

### 3.1 Structural and Compositional Analysis

**Figure 2:**
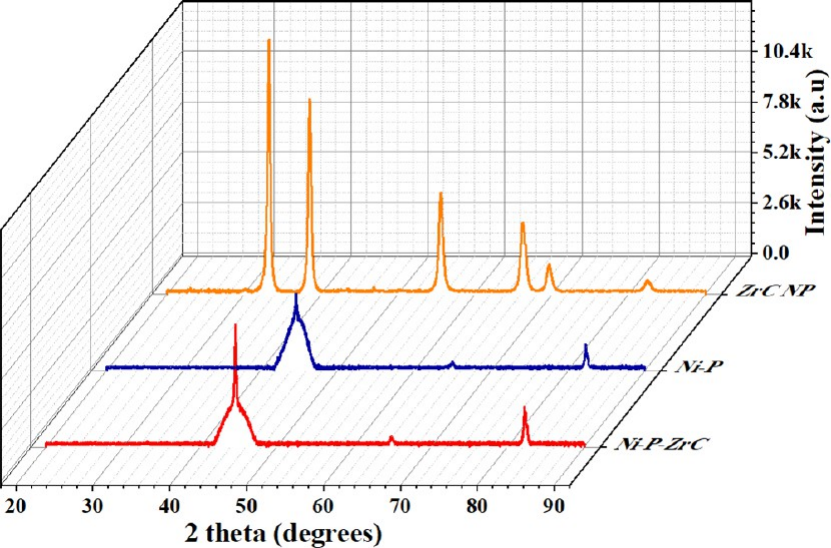
XRD diffractograms of ZrC nanoparticles, Ni-P coating, and Ni-P-ZrC nanocomposite coatings containing 0.75 g/L ZCNPs.

Structural analysis of the as-prepared Ni-P and Ni-P-ZrC metallic coatings was investigated using XRD, see Figure 2. The wide peak in the spectra of Ni-P coatings and Ni-P-ZrC nanocomposite coatings indicates the amorphous structure of the as-prepared coatings. The peak at 2θ = 45 represents a face-centered cubic lattice structure of the Ni(111) plane, which has been disturbed by the incorporation of phosphorus atoms resulting in the entire structure being amorphous, which is consistent with the previous finding.^37,52,53^ Peaks of ZrC cannot be distinguished in the spectra due to the low concentration of ZCNPs, and also, the broad peak of amorphous Ni may have shielded the peaks of ZCNPs.^19,54^ The broad peak of nickel has sharpened in Ni-P-ZrC nanocomposite coatings, which can be attributed to the presence of ZCNPs, leading to a shift in the structure from amorphous to semiamorphous.^52^ However, as a comparison, the XRD spectrum of ZCNPs shows a well-defined crystalline behavior.

### 3.2 XPS Analysis

**Figure 3:**
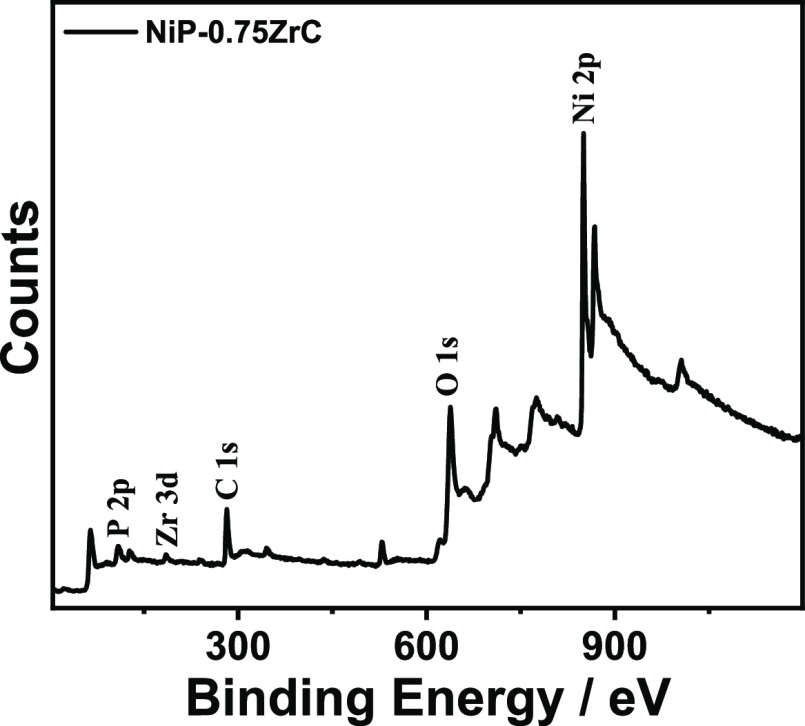
XPS survey spectrum for the Ni-P-0.75ZrC nanocomposite coating.

Figure 3 represents the XPS survey for the Ni-P-0.75ZrC nanocomposite coating, and the presence of ZCNPs in Ni-P-0.75ZrC nanocomposite coatings was confirmed from XPS analysis. The presence of the main peaks and the corresponding phases for the main elements, which correspond to Ni_2p_, O_1s_, C_1s_, Zr_3d_, and P_2p_, can be noticed. It is worth mentioning that oxygen on the coating surface could be due to the incorporation with the other elements.^55^

**Figure 4:**
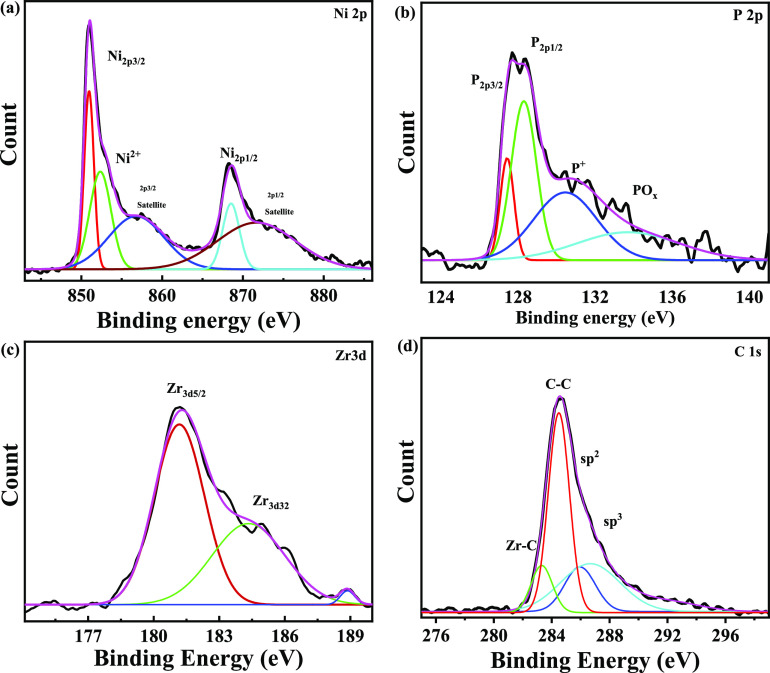
XPS spectra presenting the elemental composition of Ni-P-ZrC nanocomposite coatings, with (a) nickel (Ni_2p_), (b) phosphorus (P_2p_), (c) zirconium (Zr_3d_), and (d) metal carbide.

The individual photoionization fitted data of coating constituent elements and their respective bonding states are shown in Figure 4. High-energy resolution (HER) spectra located at 850.9 and 868.5 eV are ascribed to Ni_2p3/2_ and Ni_2p1/2_ in their metallic form, respectively, while, all the peaks positioned at 852.5, 856.8, and 871.9 eV are attributed to nickel oxide or/and nickel hydroxide of Ni_2p3/2_ and Ni_2p1/2_ chemically represented as NiO and Ni(OH)_2_, see Figure 4a, respectively. The construction of Ni(OH)_2_ and/or NiO could be ascribed to the reaction with hydroxyl ions (OH^–^) present in the aqueous solution used for electrolysis and other oxidation phenomena.^46,48^ Moreover, the peaks located at 127.4 and 128.3 eV are linked to 2p_2/3_ and 2p_1/2_, see Figure 4b. The peak of 130.5 eV can probably be attributed to (i) phosphorus hypophosphite in its elemental form or/and (ii) the phosphorus species in their intermediate forms (P(I) or/and P(III)), which remain existing inside Ni-P coatings. Nevertheless, the fitted peak at 133.8 eV corresponds to a mixture of hydroxides or/and oxides (P-OH or/and P_2_O_3_) with corresponding chemical states.^48^Figure 4c displays the high-resolution (HER) spectra of Zr 3d. The Zr 3d peaks presented at 180.4 and 183.1 clearly confirm the existence of the ZrC phase within the coating matrix.^56,57^ Furthermore, the peak situated at 282.5 eV is assigned to the Zr–C bond. Moreover, the peaks at binding energies of 284.3 and 285.2 are attributed to sp^2^ and sp^3^ hybridization of carbon, respectively.^58,59^

### 3.3 Surface Morphology

**Figure 5:**
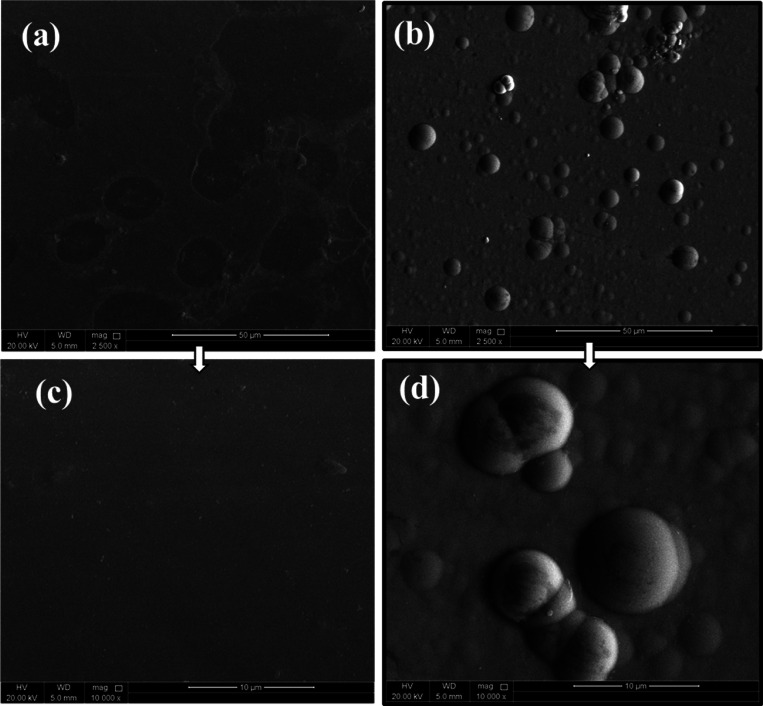
Highly magnified micrographs (FE-SEM) of developed nanocomposite coatings; Ni-P (a,c) and Ni-P-ZrC nanocomposite coatings (b,d), at two different magnifications.

AFM and FESEM were used to study the morphological and topographical characteristics of the as-prepared alloys. FE-SEM micrographs of Ni-P and Ni-P-ZrC nanocomposite coatings are depicted in Figure 5. As seen in the micrographs, Ni-P coatings (Figure 5a,c) have a plain type of structure, which is modified by the incorporation of ZCNPs. The growth of nodules is observed as a result of introducing ZCNPs in the chemical bath (Figure 5b,d). As for the Ni-P coating, a plain morphology is observed, which has changed to nodular by the addition of ZCNPs in the chemical bath, see Figure 5a,c. This can be assigned to the growth in the number of sites for nucleation of Ni and P ions, which can be deposited on the substrate owing to the large surface area of ZCNPs.^54,60,61^ Moreover, the surface of as-prepared coatings is crack-free and pore-free, indicating the good quality of the developed Ni-P and Ni-P-ZrC nanocomposite coatings. The thickness of as-prepared Ni-P alloys is around ∼12.0 μm and is achieved under the optimized experimental conditions.

**Figure 6:**
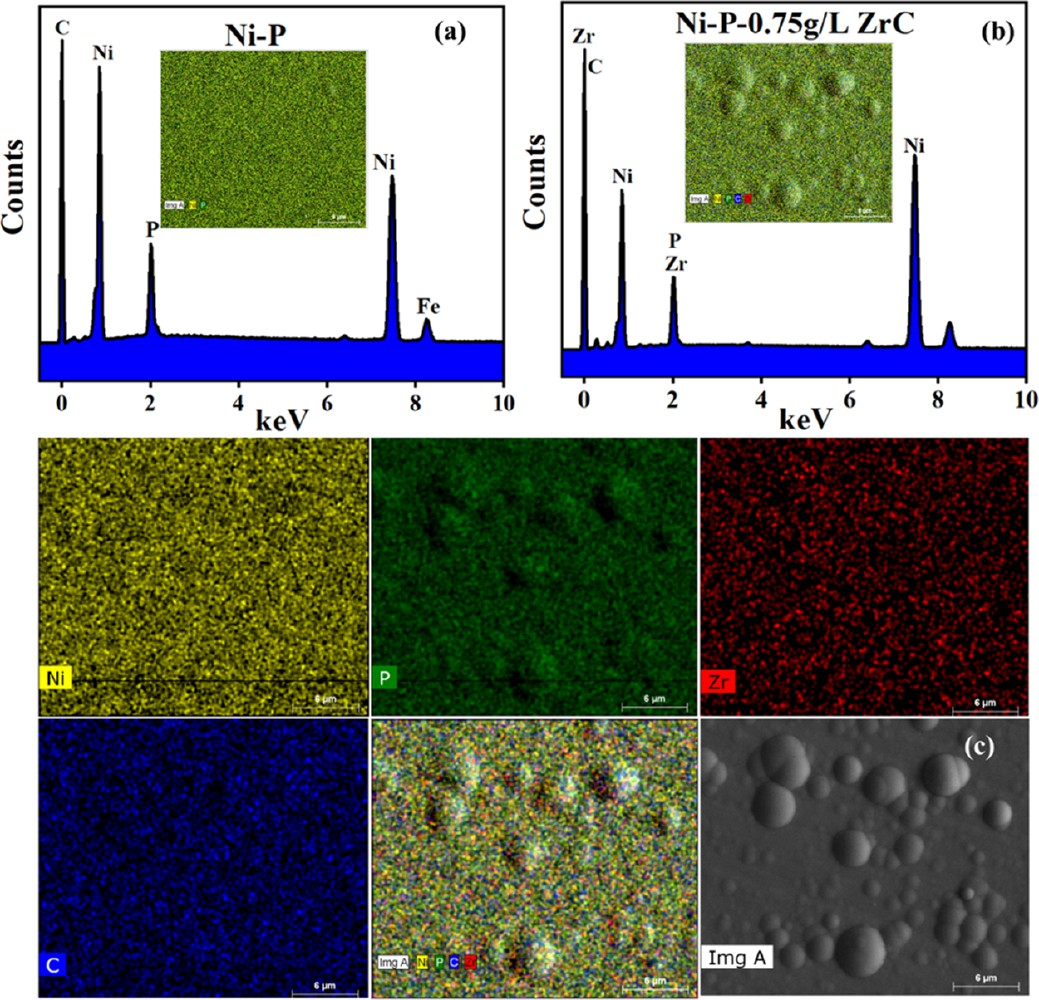
EDS elemental mapping of (a) Ni-P and (b) Ni-P-ZrC nanocomposite coatings and (c) detailed elemental mapping.

EDX analysis was further used to evaluate the presence of ZCNPs within the as-prepared alloy matrix, see Figure 6. It can be observed in Figure 6a that in the pure Ni-P coating, nickel (Ni), phosphorus (P), carbon (C), and iron (Fe) peaks are present, confirming the deposition of Ni-P. However, iron and carbon peaks could be due to the steel substrate. The presence of zirconium (Zr), carbon (C), nickel (Ni), and phosphorus (P) confirms the ZCNP inclusion into the Ni-P alloy, see Figure 6b. Additionally, the distribution of each element within Ni-P-ZrC nanocomposite coatings is also provided, indicating the uniform incorporation of ZrC nanospecies in the Ni-P matrix to form the Ni-P-ZrC nanocomposite coating, see Figure 6c.

The composition of Ni-P coating and Ni-P-ZrC nanocomposite coatings is presented in Table 2. The existence of nickel and phosphorus is evident in all the coatings in large percentages. However, a relatively higher presence of carbon can be ascribed to the inference from the substrate and surrounding environmental carbon integrated along with the occurrence of carbon from ZCNPs.^62^

**Table 2:** Quantitative Elemental Analysis of Ni-P and Ni-P-ZrC Nanocomposite Coatings

sample no.	coating composition	nickel	phosphorus	zirconium	carbon
1	Ni-P	88.62%	11.38%		
2	Ni-P-0.75 g/L ZrC	66.76%	8.18%	1.69%	23.92%

**Figure 7:**
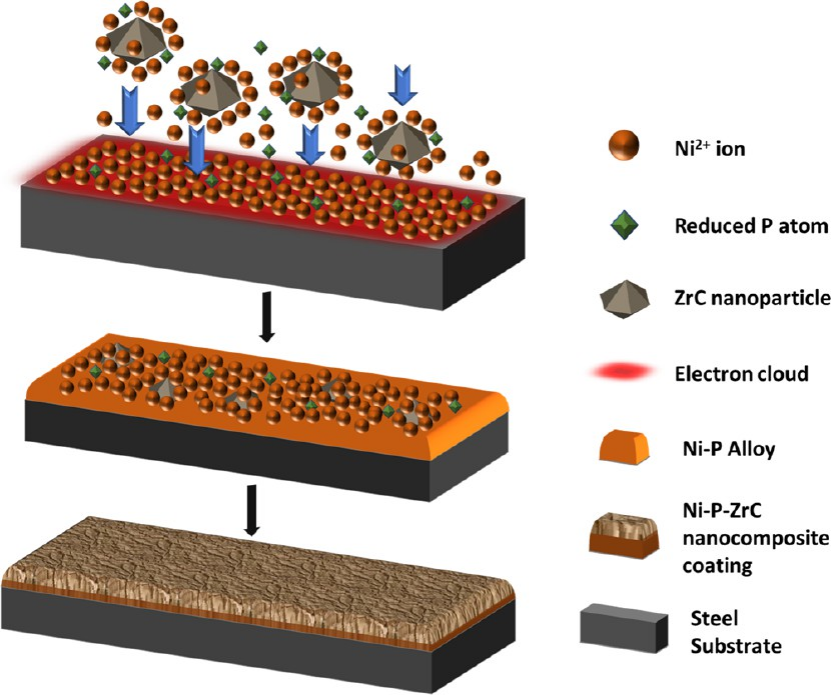
Schematic illustration of incorporation of ZrC nanoparticles at the cathode (polished steel substrate) to produce Ni-P-ZrC nanocomposite coatings.

Many researchers have suggested the codeposition process of several reinforcements within the Ni-P composite system. According to the Guglielmi model,^63^ particles first gently adsorb at the surface of the cathode through forces attributed to van der Waals attraction and then Coulomb forces responsible for heavy adsorption and bonding. This model does not account for the size of the particle and hydrodynamics of the deposition. The correction factor to resolve for the magnetic stirring was proposed by Berçot et al.^64^ Bahadormanesh and Dolati improved the original model to account for the significant percentage of the second phase deposition.^65^ Furthermore, Fransaer et al.^66^ developed a spherical particle trajectory model in which they listed out different forces acting on any spherical particle, which is revolving in a disc electrode device. Celis et al.^67^ reported that the electrodeposition process of incorporating reinforcements is said to involve five stages, including (a) reinforcement species being surrounded by an ionic cloud, (b) migration of reinforcement species directed by convective forces near the hydrodynamic layer of the cathode film, (c) adsorption of the reinforcement species accompanied by the cloud of ions at the surface of the cathode, (d) diffusion of reinforcement species by a double layer, and (e) contributing to the permanent incorporation of reinforcement species by the reduction of the ionic cloud within the alloy matrix. To summarize, the electrodeposition process requires the following step: the migration of reinforcement species to the hydrodynamic layer of the cathodic surface from the bulk of the electrolyte. Particles in this layer are attributable to forced convection and electrophoresis. Particles attached to the cathode surface owing to van der Waal forces, and finally, reduction of the ionic cloud around reinforcement species results in irreversible entrapment of the reinforcement species, see Figure 7.

**Figure 8:**
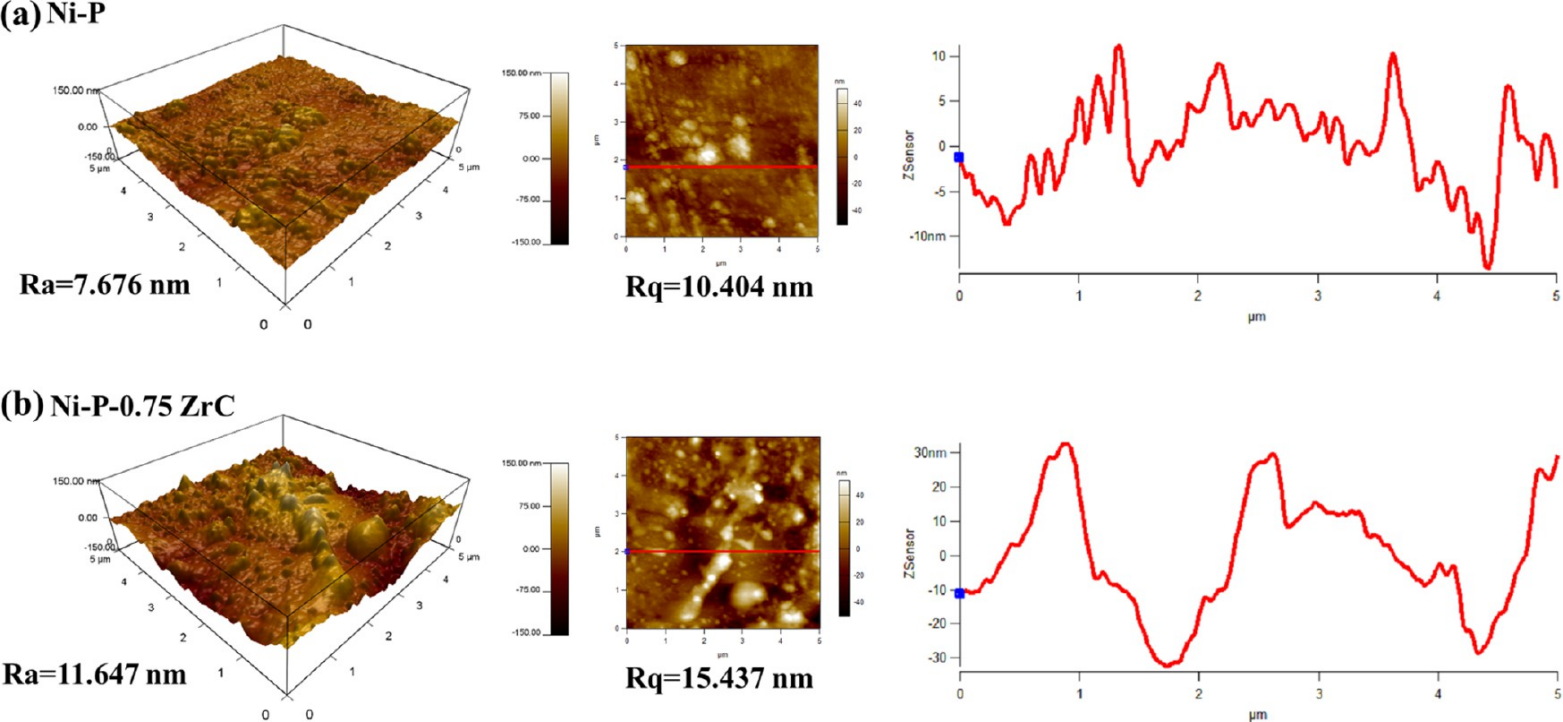
Three-dimensional AFM micrographs of as-prepared coatings and their profile of surface roughness: (a) Ni-P and (b) Ni-P-ZrC nanocomposite coatings.

A comparison of the surface topography of Ni-P and Ni-P-ZrC nanocomposite coatings is displayed in Figure 8. The incorporation of ZCNPs has enhanced the grain growth and increased the surface roughness of the coatings, which can be observed in the 3D AFM images, see Figure 8a,b. The corresponding profiles of roughness for Ni-P and Ni-P-ZrC nanocomposite coatings are also displayed for a clear comparison, see Figure 8a,b. The *R*_a_ (average roughness) of the Ni-P coating is ∼7.7 nm, which increases to 11.6 nm on the addition of 0.75 g/L ZCNPs into the matrix, which can be essentially ascribed to the existence of unsolvable and hard ceramic species into the Ni-P matrix. Moreover, *R*_q_ (RMS roughness) also increases from 10.4 to 15.4 nm for the nanocomposite coating compared to the Ni-P coating, which is in agreement with the average roughness. ZCNPs have boosted the surface roughness of the deposited coating.^46,48,54^

### 3.4 Mechanical Properties

**Figure 9:**
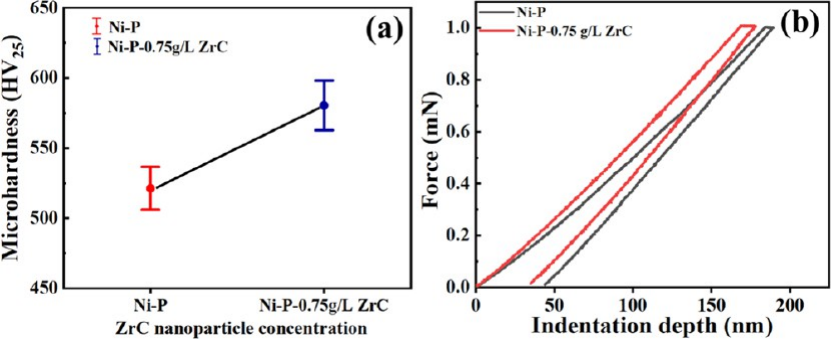
Mechanical characteristics of Ni-P and Ni-P-ZrC nanocomposite coatings; (a) Vickers microhardness and (b) load indentation depth graph of Ni-P and Ni-P-0.75ZrC nanocomposite coatings.

The mechanical properties of the prepared Ni-P coating and Ni-P-ZrC nanocomposite coating were explored by Vickers microhardness testing and nanoindentation techniques. Microhardness outcomes for Ni-P and Ni-P-ZrC nanocomposite coatings are presented in Figure 9a. The addition of ZCNPs has enhanced the coating hardness proving the classical concept of matrices and reinforcements to improve their individual properties. The Ni-P coatings demonstrate a hardness of ∼520 ± 10 HV_25_, whereas the hardness of Ni-P-ZrC nanocomposite coatings is enhanced to ∼580 ± 15 HV_25_ contributing an increase of ∼12%. This development in the hardness can be attributed to the resistance to deformation offered by high-strength ZCNPs by inhibiting the dislocation movement and restricting the plastic flow of the Ni-P matrix. It can be considered that a combination of dispersion hardening and construction of the composite structure led to the improvement in microhardness.^46,49^

Mechanical properties of the as-fabricated coatings were further analyzed through the nanoindentation technique, and the outcomes are presented in Figure 9b. It can be noted that the loading and unloading curve of Ni-P is a relatively larger area than that of Ni-P-ZrC metallic coatings. The indentation depth of the Ni-P coating decreased from ∼43.6 to ∼33.1 nm by incorporating 0.75 g/L ZCNPs, revealing an enhancement in the indentation resistance.^46,48,68^ It is noteworthy that the deficiency of discontinuity in the nanoindentation pots indicates that the as-electroplated alloys contain minimum defects (porosity, inhomogeneity, cracks, etc.).

The nanoindentation profiles were utilized for the quantitative investigation of the hardness of the as-electroplated coatings. For a clear comparison, various parameters resulting from load vs indentation depth profiles are also tabulated in Table 3. The mechanical hardness of as-prepared metallic coatings was explored using the Oliver–Pharr technique applying a maximum 1 mN indentation force through the Berkovich diamond indenter. The loading and unloading rate was set at 200 μN/s; meanwhile, a dwell time of 5 s was fixed at a full load. The hardness of the Ni-P alloy improved from 4.98 to 5.75 GPa upon the addition of 0.75 g L^–1^ ZCNPs. The presence of ZrC nanospecies in the Ni-P alloy obstructs the movement of the dislocations leading to the development of the mechanical properties of the Ni-P-ZrC nanocomposite coatings. Similarly, stiffness of the Ni-P-0.75ZrC nanocomposite coating is observed to increase from 7.49 for the Ni-P alloy to 7.90 kN/m, indicating that an improvement in the deformation resistance was due to the incidence of ZCNPs in the Ni-P matrix within the elastic limit. Moreover, the modulus of elasticity of the Ni-P alloy is boosted from 14.1 to 15.8 GPa by the incorporation of 0.75 g/L ZCNPs.^69^

**Table 3:** Derived Parameters from Load Indentation Profiles of Ni-P and Ni-P-ZrC Nanocomposite Coatings

sample no.	composition	elasticity (GPa)	stiffness (kN/m)	hardness (GPa)
1	Ni-P	14.05	7.49	4.98
2	Ni-P-ZrC	15.88	7.90	5.75

### 3.5 Wear Test

**Figure 10:**
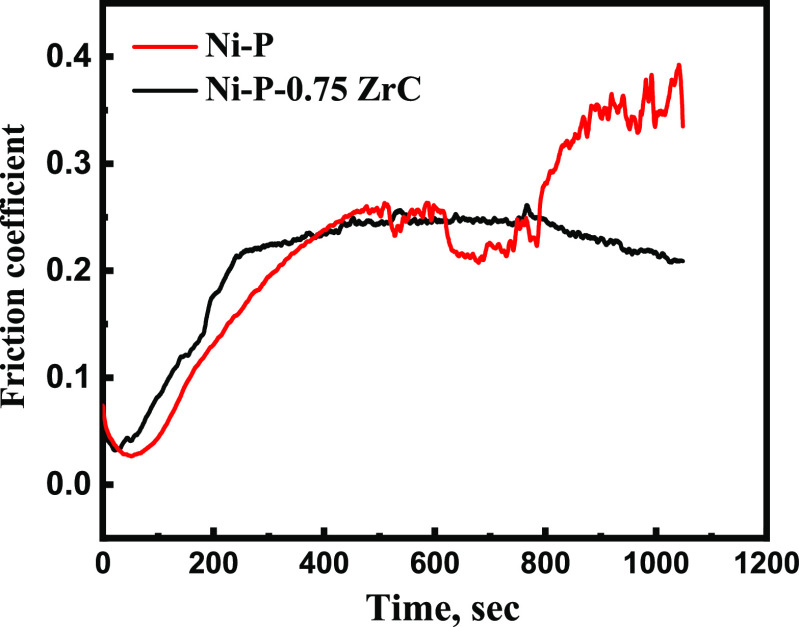
Wear test of the as-electrodeposited nanocomposite coatings prior to and after the incorporation of ZrC nanospecies.

The coefficient of friction (COF) as a function of time for the coelectroplated Ni-P and Ni-P-0.75ZrC nanocomposite coatings is displayed in Figure 10. The friction coefficient decreased from 0.34 for electrodeposited Ni-P to 0.2 after 0.75 g/L ZrC was incorporated in the Ni-P matrix. The COF was boosted at the initial stage of the friction time due to contact friction between the protruding part of the as-electroplated substrates and the stainless-steel ball. The COF of the metallic Ni-P coating oscillated after 400 s and significantly increased to a higher value after 800 s, which could be ascribed to the coating removed from the substrate resulting from destruction (shear) of attachment between the metallic alloy and counterface asperities. It is noteworthy that the presence of COF fluctuation can be categorized among vast and brief domains. These instabilities could be ascribed to the ejection and accretion of the wear debris.^49,68−70^ On the contrary, in the case of Ni-P-ZrC, a smooth and constant COF was observed after 200 s of friction time, which could be attributed to the lubrication influence of the nanospecies.

**Figure 11:**
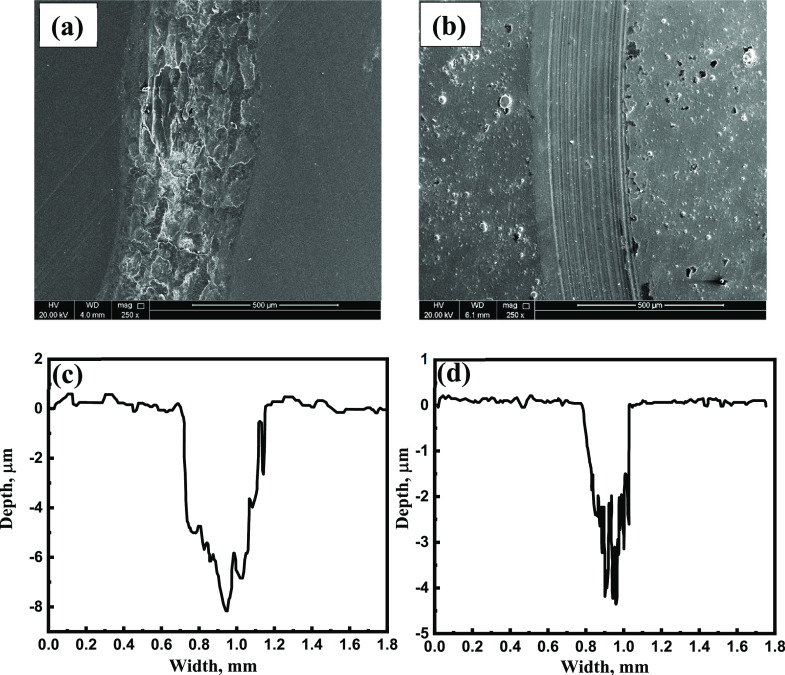
SEM images of (a) Ni-P and (b) Ni-P-0.75 g/L ZrC after wear tests with their wear depth profile (c,d).

The wear rate (*w*_s_) of the metallic Ni-P coatings prior to and after incorporation of ZrC is calculated by the following equation.^71^
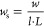
1where *w* is the loss of weight (g), *l* represents total sliding displacement (m), and *L* is attributed to the load applied (N) during the test. The wear rate (*w*_s_) of Ni-P is lessened from ∼89 to 38 μg N^–1^ m^–1^ due to the incorporation of ZrC nanospecies. Moreover, the wear track and depth of Ni-P alleviated from 456 and 8.1 μm to 295 and 4.4 μm as a result of the incorporation of 0.75 g/L ZrC nanoparticles (Figure 11c,d).

**Figure 12:**
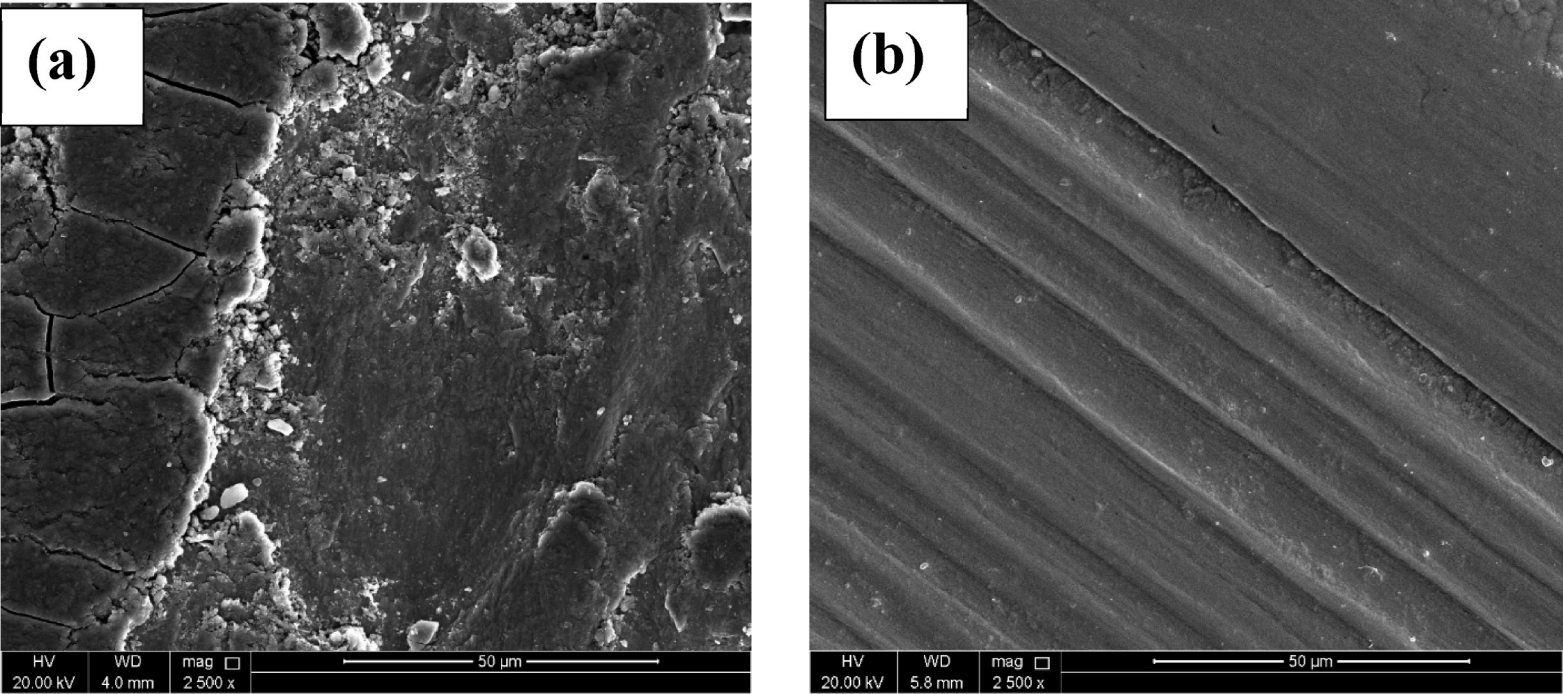
Highly magnified SEM images of wear track areas of (a) Ni-P and (b) Ni-P/0.75ZrC metallic alloy.

Figure 12a illustrates the SEM image of the worn surface of the Ni-P metallic coatings at higher magnification. The formation of fatigue microcracks in the Ni-P metallic coating as a result of inherent properties, such as low hardness, ductility, an apparent poor adhesion, and internal stress in the coating matrix, can be observed. The attendance of cavities or grooves could be attributed to the removal of tribolayers or surface oxide layers, aiding slipping wear by resting with the worn metallic coatings and abrasive as a separate identity.^71^ Accordingly, the wear regime in Ni-P coatings is adhesive. Figure 12b shows characteristic plowing furrows without any visible microcracks, which are attributed to the enhanced hardness value of nanocomposite coatings. The incorporation of ZCNPs into Ni-P exhibits linear wear tracks, indicating an abrasive wear approach.^72^

### 3.6 Erosion Test

**Figure 13:**
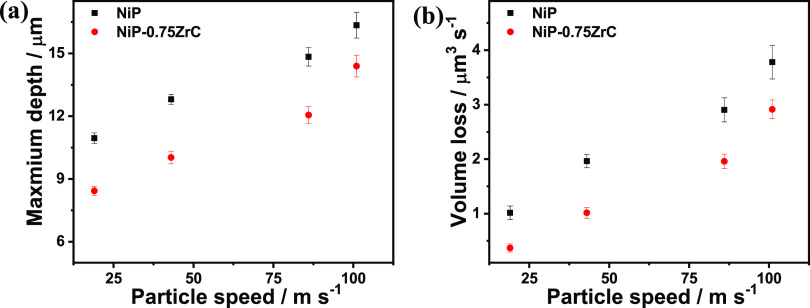
(a) Maximum erodent depth and (b) volume loss for the Ni-P and Ni-P-0.75ZrC nanocomposite coatings at different particle velocities after 30 s of erosion time.

Figure 13a represents the maximum measured depth versus particle speed dependence at the same exposure time. It can be estimated that the depth is proportional to the particles’ velocity, indicating higher coating loss at a higher speed. Moreover, the maximum erosion depth is lessened from 16.3 to 13.5 μm with amending 0.75 g/L ZrC to the coating matrix at 101 m s^–1^. In the meantime, Figure 13b depicts the volume loss of Ni-P and Ni-P-0.75ZrC nanocomposite coatings at different speeds of the erodent particles. The volume loss rate is derived from the average erosion depth and the measured eroded area per exposure time. As expected, the Ni-P-0.75ZrC nanocomposite coatings have better erosion resistance than the Ni-P coating. Moreover, the volume loss rate at 19 m s^–1^ diminished from 1.23 to 0.38 μm^3^ s^–1^ for Ni-P and Ni-P-0.75ZrC alloys, respectively, indicating that damage in the Ni-P-0.75ZrC coating is three times lower than that of the bare coating at low erodent speed. Meanwhile, the volume loss rate at 101 m s^–1^ of the erodent particles is reduced from 3.7 to 2.9 μm^3^ s^–1^ for Ni-P and Ni-P-0.75ZrC coatings.

**Figure 14:**
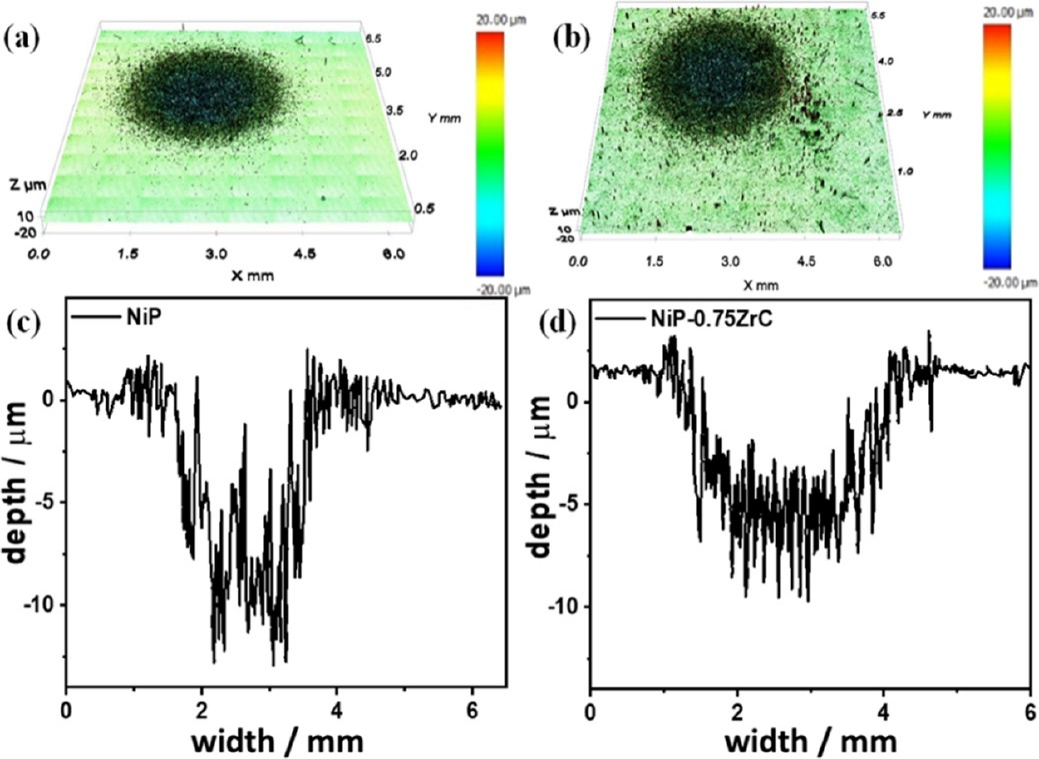
Topographic images of (a) Ni-P and (b) Ni-P-0.75ZrC and their respective depth and width profile (c,d) after 30 s of erosion time at a 101 m/s particle velocity.

Figure 14 depicts the optical profilometry of the eroded substrates of the Ni-P and Ni-P-0.75ZrC nanocomposite coatings after 30 s of erosion time at 101 m s^–1^. It can be seen that the surface roughness for the Ni-P coating is lower when compared to as-synthesized Ni-P-0.75ZrC nanocomposite coatings after erosion tests, as seen in Figure 14a,b. Additionally, the penetration depth of the Ni-P alloy is higher than that of the Ni-P-0.75ZrC nanocomposite coating, as demonstrated in Figure 14c,d.

### 3.7 Corrosion Studies

#### 3.7.1 Electrochemical Impedance Spectroscopy (EIS)

**Figure 15:**
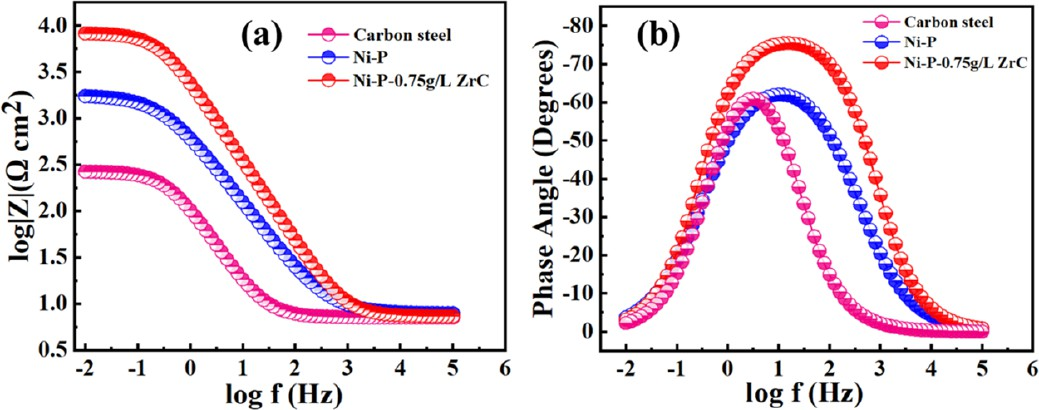
(a) Bode plot of the polished carbon steel, Ni-P, and Ni-P-0.75 g/L-ZrC nanocomposite coatings containing the frequency impedance magnitude curve and (b) frequency phase angle curve after 2 h of immersion in 3.5 wt % NaCl solution.

**Figure 16:**
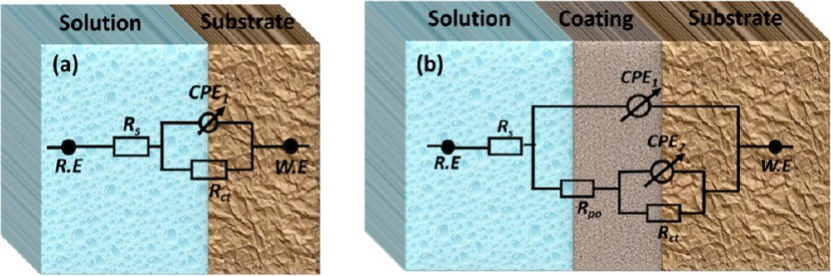
Equivalent electric circuits applied to fit EIS data for (a) substrate and (b) Ni-P and Ni-P-0.75ZrC nanocomposite coatings.

EIS is a widely accepted method for exploring the corrosion mitigation of the as-fabricated coatings. EIS graphs of the polished carbon steel, Ni-P, and Ni-P-0.75ZrC nanocomposite coatings are shown in Figure 15. Experimental data for the substrate were fitted using the modified version of the Randle cell in which a pure capacitor was improvised with a constant phase element to account for the pure capacitance, as shown in Figure 16a. For describing the degradation behavior of Ni-P and Ni-P-ZrC nanocomposite coatings, a two-time constant cascaded electrical equivalent circuit was employed to fit the EIS data set, as shown in Figure 16b. The electric circuits consist of *R*_s_ for the resistance of the brine solution used for the test, whereas *R*_po_ and *R*_ct_ account for the resistance due to pores and resistance due to transfer of charge of coatings. Constant phase elements (CPE_1_ and CPE_2_) were utilized instead of a pure capacitor to justify the discrepancy at the surface and interface of the metallic coating computed from the following equation^46^
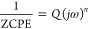
2in which *Q* stands for admittance, ω is the angular frequency, and *n* is the exponent for the constant phase element, which is responsible for the nature of capacitance such that closer to unity means a pure capacitor.

The Bode plots of the polished steel substrate, Ni-P coating, and nanocomposite coating are presented in Figure 15. It can be perceived that the corrosion resistance of the polished steel substrate is relatively small (260 Ω cm^2^). Ni-P coatings possess more corrosion resistance than carbon steel as the impedance value of the Ni-P coating is 1782.8 Ω cm^2^, which can be ascribed to the construction of a protective film of hypophosphite as a result of the electrochemical reaction of saline solution with the Ni-P coating.^54,73^ Further incorporation of secondary phase ZCNPs in the Ni-P matrix changed the impedance response, resulting in widening of the phase angle plot. This implies an additional protective nanocomposite coating (move toward higher frequencies) and, conversely, the existence of other activities (reduced corrosion).^48,49,71^ The improvement in the impedance of nanocomposite coatings can be attributed to reducing the dynamic corrosion sites due to the trapping of hard, inactive, and corrosion-resistant ZrC nanoparticles. Interestingly, the incorporation of 0.75 g/L ZrC nanoparticles increased the *R*_ct_ value to 8353 Ω cm^2^, which is four times higher than that of the Ni-P alloy.

**Figure 17:**
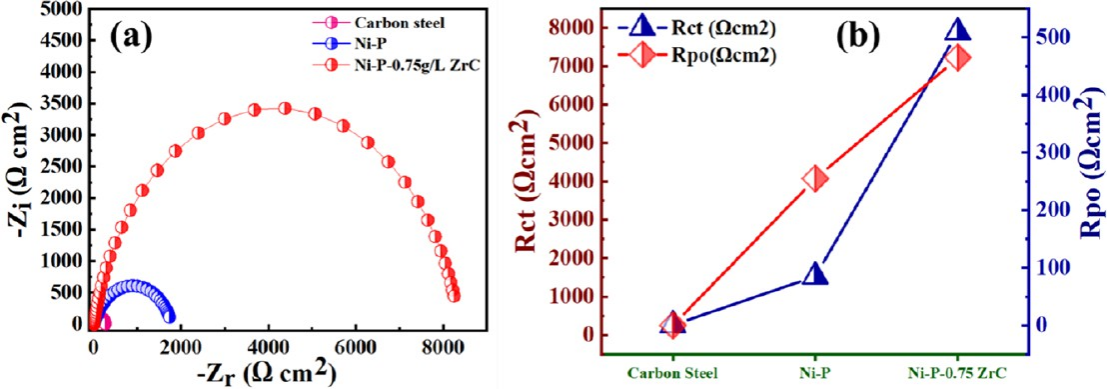
(a) Nyquist plot for carbon steel and the as-fabricated Ni-P and Ni-P-0.75ZrC nanocomposite coatings in 3.5 wt % NaCl solution and (b) variation of *R*_po_ and *R*_ct_ on the substrate, Ni-P, and Ni-P-0.75ZrC nanocomposite coatings.

Figure 17a shows the Nyquist plots for the polished carbon steel substrate, Ni-P, and Ni-P-0.75ZrC nanocomposite coatings. The two-time constant equivalent circuit was used to fit experimental data as exhibited in Figure 16b. The semicircular radius of the Nyquist curve reveals a successive increase, pointing to high corrosion impedance resulting from incorporating ZrC nanoparticles. The incorporation of ZrC nanospecies in the Ni-P alloy increased the polarization and pore resistance of the as-fabricated coatings, see Figure 17b. Enhancement in the corrosion resistance of the Ni-P alloy as a result of reinforcing the inert ZrC nanospecies that fill the defects existing in the Ni-P matrix such as pores and microcracks leads to a burden in the entrance of the hydrated Cl^–^ species to reach the carbon steel surface.^46,48,71^

#### 3.7.2 Tafel Test

**Figure 18:**
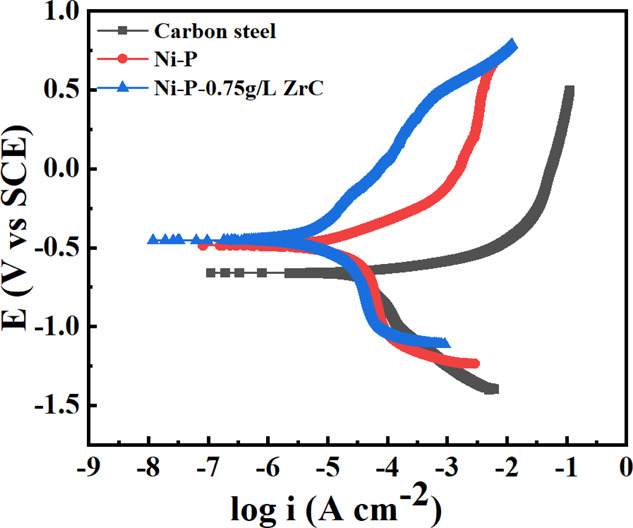
Tafel plots of polished carbon steel, Ni-P coating, and Ni-P-0.75ZrC nanocomposite coating in 3.5 wt % NaCl solution.

Tafel polarization was further utilized to evaluate the corrosion resistance of the polished steel substrate, pure Ni-P coating, and as-prepared Ni-P-ZrC nanocomposite coatings with 0.75 g/L ZrC nanoparticles by setting the rate of scan at 1 mV s^–1^ described in Figure 18. Electrochemical corrosion currents (*i*_corr_) were acquired from the fitted curve and are presented in Table 4. In addition, the efficiency of corrosion protection (PE%) was estimated utilizing the following formulation^71^
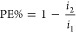
3where *i*_1_ is the current density of the polished steel substrate and *i*_2_ is the current density of coated samples. Polished carbon steel is observed to have the highest current density of 56.9 μA cm^–2^ with an electrode potential of 658 mV. However, the maximum value of current density for the Ni-P coating is observed to be 16.5 μA cm^–2^ at a potential of 486 mV, showing development in the corrosion resistance of 71.03%. On the other hand, the incorporation of 0.75 g/L ZCNPs considerably alleviated the *i*_corr_ to 8.3 μA cm^–2^ with almost 85.4% development in the corrosion resistance.

**Table 4:** Comparison of the Corrosion Protection Efficiency (PE%) Obtained from *i*_corr_ for the Current Study and Numerous Reported Results from the Literature

sample no.	sample	deposition technique	*i*_corr_ (μA cm^–2^)	PE%	ref.
1	polished steel	electrodeposition	56.86		current study
	Ni-P		16.47	71%	
	Ni-P-0.75 g/L ZrC		8.3	85%	
2	uncoated steel	electrodeposition	39.3		(48)
	Ni-P		28.6	28.4%	
	Ni-P-Y_2_O_3_-0.25 g/L		19.6	49.1%	
	Ni-P-Y_2_O_3_-0.5 g/L		15.4	61.5%	
	Ni-P-Y_2_O_3_-0.75 g/L		8.9	77.7%	
	Ni-P-Y_2_O_3_-1.0 g/L		3.7	90.8%	
3	carbon steel	electrodeposition	55.94		(47)
	Ni–P		38.43	31.3%	
	Ni–P 0.5 g L^–1^ TiC		25.62	54.2%	
	Ni–P 1.0 g L^–1^ TiC		7.79	86.0%	
	Ni–P 1.5 g L^–1^ TiC		6.49	88.4%	
	Ni–P 2.0 g L^–1^ TiC		4.91	91.2%	
4	mild steel	electrodeposition	79		(49)
	Ni–B		50	36%	
	Ni–P		31	60%	
	Ni–B/Ni–P		17	78%	
	Ni–B/Ni–P–CeO_2_		7.5	90%	
5	HSLA steel	pulse electrodeposition	50		(76)
	Ni–P		30	40%	
	Ni–P/0.25TiC		16	68%	
	Ni–P/0.50TiC		7	86%	
	Ni–P/0.75TiC		3	94%	
6	substrate	electroless deposition	40.3		(73)
	Ni-P coating		7.1	74.8%	
	duplex Ni-P-ZrO_2_ coating		3.717	87.8%	
7	substrate	electroless deposition	8.0		(77)
	Ni–P coating		4.0	50%	
	Ni–P–WC coating		1.0	87.5%	
8	uncoated	electroless deposition	20.3		(78)
	Ni-P		4.17	79.5%	
	Ni-P CNT 0.25		3.28	83.8%	
	Ni-P CNT 0.5		2.38	88.3%	
	Ni-P CNT 1.0		2.01	90.1%	
9	Ni–P (as-deposited)	electrodeposition	177	86.7%	(79)
	Ni–P–C (as-deposited)		170	87.3%	
	Ni–P (heat-treated)		139	89.6%	
	Ni–P–C (heat-treated)		88	93.3%	
10	substrate	electroless deposition	2.65		(80)
	Ni–P (used bath A)		0.47	82%	
	Ni–P (used bath B)		0.58	78%	
	Ni–P (used bath C)		0.65	75%	

**Figure 19:**
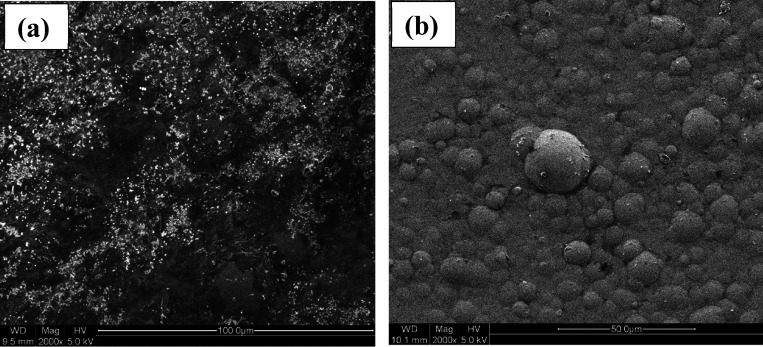
SEM images of (a) Ni-P and (b) Ni-P-0.75ZrC after corrosion tests in saline water.

Figure 19 shows the SEM images of (a) Ni-P and (b) Ni-P-0.75ZrC after corrosion tests in 3.5 wt % NaCl solution. It can be observed that the Ni-P coating is significantly corroded; however, Ni-P-0.75ZrC exhibited a slight influence on the deposited coating with the formation of few pits. The improvement can be associated with incorporating ZCNPs in the Ni-P coating matrix, which has enhanced corrosion mitigation after the addition of the ZCNPs by reducing the active positions for the adsorption of Cl^–^ ions on the flaws of coatings such as pores and cracks.^74,75^

Table 4 compares the protection efficiency of the as-electrodeposited Ni-P-0.75ZrC with the reported nanocomposite coatings in the literature.

## 4 Conclusions

Ni-P and Ni-P-ZrC nanocomposite coatings containing 0.75 g/L ZrC nanoparticles (ZCNPs) were successfully developed through the electrodeposition technique. A comparison of structural, surface, mechanical (hardness, nanoindentation, wear, and erosion), and electrochemical properties (corrosion resistance) indicates that Ni-P-0.75ZrC nanocomposite coatings demonstrate improved properties when compared with Ni-P coatings. The improvement in mechanical properties can be ascribed to the dispersion hardening effect and formation of a composite structure. The enhancement in the corrosion resistance properties can be regarded as the effect of reduction in the active area of the Ni-P matrix by the presence of inactive ZCNPs. The tempting properties of Ni-P-0.75ZrC nanocomposite coatings provide their potential application in various industries.
